# Multiple Neuritis

**Published:** 1887-08

**Authors:** T. M. Holmes

**Affiliations:** A. A. Surg. U. S. M. H. S., Rome, Ga.


### MULTIPLE NEURITIS.

BY T. M. HOLMES, M. D., A. A. SURG. U. S. M. H. S., ROME, GA.

G. H. M., æt. 22, a Georgian, made application, October 18, 1886, for admission into the U. S. Marine Hospital, at this port, with the following history: He had been for three years acting as clerk upon the steamboats plying the Coosa river. The nature of the position was such as to subject him to much exposure, especially during the winter months, when freights were heavy and the atmospheric changes extreme, and being of an imprudent disposition, he was constantly exposing himself unnecessarily, going frequently during the coldest weather without his coat, and at times, when very tired, falling asleep in the open air, and on awaking would be so stiff and cold as to require much rubbing before being able to move.

Moreover, owing to the influence to which all sea-faring men are subject in a greater or less degree, he had fallen into intemperance, and though he probably had never been under the influence of alcoholic spirits in an extreme degree, yet he came under that large and unfortunate class commonly denominated "topers." As a natural result of this, he had become much bloated and was a chronic dyspeptic. Again, about five years ago, he was the subject of a venereal sore, and was treated by the town charlatan for syphilis.

For some weeks prior to the sudden onset of his trouble, he had been complaining of persistent pains in the muscles of his arms, back and legs, which seemed to be of a rheumatic character. For these I had been treating him in the out-patient department with salicylate of soda, but with only partial relief, when, without any other prodromic symptoms, he experienced an inability to control his lower limbs. He had started to ascend the steps leading to the upper deck of the boat, when suddenly his legs gave way and he fell to the floor. He was immediately carried to the hospital, where I saw him the following day.

I found him in the recumbent position, with an utter inability to move himself. He had "drop-wrist" and "drop-feet," and had no control of the muscles of volition. He could not change his arms or legs from any position into which they might helplessly fall. Every muscle in the body seemed to share in this general paralysis. The diaphragm was apparently totally paralyzed, causing the respiration to be entirely thoracic, about thirty per minute, deep and labored. The pulse was quick and small, and numbered 130 to 135 per minute. Hyperesthæsia and hyperalgæsia were prominent symptoms, it being almost impossible to even touch him, as in the changing of his position for his comfort, without giving him great pain. There were constant and almost unbearable pains in his feet and hands, characterized by a tingling sensation, paræsthesæ and formication. These pains were so great as to produce insomnia, and these *two* symptoms were the bane of his existence, from which he never failed to pray me for relief. They were also to him unfailing barometers, always increasing in intensity a few hours antecedent to a change of the atmosphere to a state of greater humidity. His extremities were warm to the touch, but warm applications would relieve the pains momentarily. There was almost continually an expression on the patient's face that betrayed his inner feelings of discomfort. Every muscle in the body was a "touch-me-not," which, when pressed upon, caused the patient to cry. Delayed sensation, too, was a prominent symptom ; a pin being stuck into him could not be felt for some seconds. The "tendon reflex" was totally absent, and he was troubled with diplopia for several days after he was prostrated—objects on the wall appearing double. He was utterly without strength in any way whatever, even the grasp of his hand being scarcely perceptible. His appetite was at first somewhat impaired, but it gradually grew to be enormous, and, strange to say, his digestion improved correspondingly. His bowels, however, were constipated, and he voided his urine only at long intervals. This, I think, was due to as many as three causes: (1) the lack of nervous force; (2) the effect of the opiates necessary for the relief of pain, and (3) to the dread incident to his utter helplessness. Besides these annoyances, profuse night-sweats added greatly to his discomfort. His brain evidently shared to a certain extent in the general nervous derangement, as his memory was much impaired. He had no idea of time. On being asked, about the sixth week of his illness, how long he had been in bed, he replied, "About a week." He frequently repeated a question to me the third or fourth time during one of my visits with as much complacency as at first. After several months of his illness, when his condition was much improved, he discovered that the middle finger of his left hand, on being elevated a little, would enter into the most amusing gesticulations, resembling very much the actions of a little automatic limber-jack. He was very fond of showing this to every visitor, and sometimes did so with equal pleasure two or three times during a single visit, making the same comments upon it each time.

As the weeks passed the most of his symptoms improved slowly but perceptibly. There were two prominent exceptions, the atrophy of the muscles and the drawing of the muscular tendons, causing the arms and legs to become flat and thin, and the fingers and toes to be flexed to their utmost. The nails became hard and brittle, and became thick in the middle, tapering toward their ends, presenting a falcon-like appearance—the so-called "hob-nail." The wrist, elbow, ankle and knee-joints were in the same flexed and fixed condition that the fingers and toes were. For weeks after his confinement, this general tendency to contraction caused the feet and legs to draw up in bed, and he had no power to force them down again. An attempt to correct this deformity in any of the parts forcibly gave great pain.

Dr. M. Allen Starr (whose graphic lectures this winter before the New York Pathological Society, on multiple neuritis, were read with so much interest) divides the etiology of this disease into three distinct classes: (1) "Toxic cases, due to poisoning by alcohol, arsenic and bi-sulphide of carbon;" (2) "Infectious cases, due to the direct action upon the nervous system of the infectious agents, producing diphtheria, variola, typhoid and typhus fevers, severe malarial fever and tuberculosis, to which must be added the agent causing the epidemic form of neuritis known as kake or beriberi;" (3) "Spontaneous causes, due to uncertain causes, among which cold and exposure to damp and wet, and to over-exertion, may find a place." I feel satisfied that alcohol and exposure to cold were the prime, if not the sole, factors in the production of this case.

The literature of multiple neuritis is of such recent date that but little is known of the disease in this country. It has always been confounded with locomotar ataxia and other paralyses, and not until lately has its pathology been truly understood, and the disease given its proper and distinctive place in the nosology of diseases. Instead of being a disease of the spinal cord, as one would naturally suppose from the universal paralysis, the pathologists of recent years have demonstratcd the fact that the pathological condition producing this and other symptoms really exists in the peripheral nerves. On dissecting the nerves of subjects that have died with the above symptoms, and subjecting them to a careful microscopical examination, they have been found to be in various stages of degeneration. The axis-cylinders near the nerve roots have been found increased in number or entirely destroyed, the medulary sheaths being broken up into fatty fragments, the myclin dividing into large and small drops containing fat granules, and the degenerative mass within the schwann sheath consisting of myclin and axis-cylinder has been found in various stages of absorption, the conditions varying in different subjects (Starr). Sometimes only certain peripheral nerves are affected. In this case, we have unmistakable evidence that the sensory, motor and the involunary nerves, to a certain extent, were implicated—the general paralysis, perverted sensation and condition of respiratory organs amply attesting this fact.

### TREATMENT.

During the first three months, iodide of potash and comp. tincture of gentian with digestives constituted the chief therapeutical agents. The iodide was given at first in doses of ten grains, three times a day, and gradually increased to one hundred grains a day, this maximum quantity being kept up for two or three weeks, and then dropped entirely for a week or ten days to give the stomach and heart a rest. Opiates were administered when absolutely necessary for the relief of pain and insomnia. This was done hypodermically, as it was soon discovered that when given any other way, it required several hours to secure their good results. After weeks of the above constitutional treatment, elixir of phosphate of iron, quinine and strychnia and the syrup of hypophosphites compound were faithfully tried. Besides these efficient nerve foods, the constant electrical current was frequently employed. At first this gave great pain, but failed to produce the natural muscular contraction, the whole muscular mass of any part seeming to be one soft, flabby muscle without tonicity. Whisky in moderate quantities was administered at regular intervals as a stomachic and cardiac stimulant, and the extracts of malt and wine of coca were alternately employed. After the hyperesthesia and hyperalgæsia had abated to a great extent, massage constituted the main treatment. This was pushed vigorously, and was followed by the most satisfactory results. The thin arms and legs soon began to thicken and increase in volume, so that within a few weeks they would not have been recognized as the same limbs. These are now almost naturally straight, the yet partially contracted fingers and toes constituting the patient's only deformity. He is perfectly free of pain, is quite fleshy, has a clear, fresh, and healthy complexion, is able to raise up and sit on the edge of his bed at pleasure, and with the auspicious prospects for his continued improvement, we have every reason to believe that he will be entirely well within a few months.

